# SARS-CoV-2 Serological Surveillance of Both Vaccinated and Unvaccinated Zoo Animals with the Identification of a Sloth Bear and a Tapir with Previous Infection

**DOI:** 10.3390/v17111459

**Published:** 2025-10-31

**Authors:** Marie Arvidson, Yashaswi Raj Subedi, Sandipty Kayastha, Angel Mitchell, Kami Alvarado, Xufang Deng, Karen Terio, Matthew Allender, Leyi Wang

**Affiliations:** 1 Veterinary Diagnostic Laboratory, Department of Veterinary Clinical Medicine, College of Veterinary Medicine, University of Illinois, Urbana, IL 61802, USA; marie.arvidson@gmail.com (M.A.);; 2 Department of Physiological Sciences, College of Veterinary Medicine, Oklahoma State University, Stillwater, OK 74078, USA; ysubedi@okstate.edu (Y.R.S.); xufang.deng@okstate.edu (X.D.); 3 Fort Wayne Zoo, Fort Wayne, IN 46808, USAkami.alvarado@fwzoo.com (K.A.); 4 Zoological Pathology Program, University of Illinois College of Veterinary Medicine, Urbana, IL 61802, USA; kterio@illinois.edu; 5 Wildlife Epidemiology Laboratory, University of Illinois College of Veterinary Medicine, Urbana, IL 61802, USA; 6 Brookfield Zoo Chicago, Brookfield, IL 60513, USA

**Keywords:** SARS-CoV-2, COVID-19, serology, ELISA, zoo animals, sloth bear, tapir

## Abstract

Since its discovery in 2019, SARS-CoV-2 has continued to be detected in both humans and animals worldwide. Currently there is limited research focusing on serological surveillance of wildlife under human care. Here we tested 230 serum samples of 134 animals from two zoological institutions collected between 2015 and 2024. To assess prior exposure and antibody responses from natural infection or vaccination, we used three serological assays: a nucleocapsid protein-based ELISA (N-ELISA), a surrogate virus neutralization test (sVNT) for spike (S) protein and a neutralization assay with S-pseudotyped viral particles. Among the 114 samples collected from 58 animals at Fort Wayne Zoo in Indiana, 37 samples from 20 vaccinated animals were sVNT-positive, and 2 of the positive animals had 2 samples prior to vaccination that tested positive by N-ELISA. Of the 116 samples from 76 animals at Brookfield Zoo in Illinois, 20 samples of 20 animals were sVNT-positive, and 19 of the positive animals had been vaccinated. Among these 20 sVNT-positive samples, only one sample from a South American Tapir was positive from prior to vaccination and 1 sample from a sloth bear was also positive by N-ELISA, marking the first documented cases of SARS-CoV-2 exposure in both species. Neutralization assays with S-pseudotyped virus revealed that some of the sVNT-positive samples have strong activity against the WH1-S pseudovirus but showed significantly reduced neutralization against the Omicron LP.8.1-S pseudovirus. These results underscore the need for updated vaccines tailored to emerging variants. Overall, our findings highlight the importance of continued serological surveillance across multiple species to detect new SARS-CoV-2 exposures and monitor vaccine-induced immunity in captive animal populations.

## 1. Introduction

As the causative agent of coronavirus disease 2019 (COVID-19), severe acute respiratory syndrome coronavirus 2 (SARS-CoV-2) is responsible for a worldwide pandemic after its emergence in late 2019 and continues to be a worldwide health concern [1]. In addition to the millions of infections and deaths of human life, SARS-CoV-2 infection has been detected in many companion, agricultural, and wildlife species [2,3]. Animals infected with SARS-CoV-2 may exhibit clinical signs, such as fever, coughing, lethargy, discharge from the eyes or nose, difficulty breathing or shortness of breath, vomiting, and diarrhea [4]. There is evidence of COVID-19 having a zoonotic origin. A bat coronavirus RaTG13 strain was discovered in China and shares the highest nucleotide sequence identity of 96.1% with SARS-CoV-2 [5]. Subsequently, another bat-derived coronavirus, BANAL-20-52 (BANAL-52), was discovered in Malayan horseshoe bats (*Rhinolophus malayanus*) from northern Laos, sharing an even higher level nucleotide homology (96.8%) with SARS-CoV-2 and amino acid homology (98%) with the SARS-CoV-2 spike (S) protein [6,7]. However, there is less conservation of the S1 and amino-terminal domain genomes in these bat coronaviruses, supporting that SARS-CoV-2 might have a mosaic genome and could be derived from a recombination of several donor viruses, including RaTG13 and BANAL-52 [6].

Because of this hypothesized animal origin, there is a growing concern about wide-reaching animal susceptibility to SARS-CoV-2, as it has been shown to infect animals via natural or experimental infections [2]. At least 20 zoological animal species have been reported positive for SARS-CoV-2 in different countries [2]. In March 2020, 5 tigers and 3 lions at the Bronx Zoo were confirmed with SARS-CoV-2 infection, and further sequence analysis suggested that the tigers could have been exposed to the virus through infected zookeepers [8]. This marked the first documented reverse zoonosis (human-to-animal) SARS-CoV-2 transmission in a nondomestic feline. Since then, reverse zoonotic events have been reported globally in tigers [8,9,10], lions [9,11,12], and Canadian lynx (*Lynx canadensis*) [9]. In addition, studies reported that infected animals such as lions, cats, hamsters, and minks could be a source for secondary spillover of SARS-CoV-2 back to humans [13,14,15,16]. These findings highlight the potential risk to public health, especially if the virus evolves through adaptive mutations or recombination. Thus, protecting captive animals from the infection is a critical component of SARS-CoV-2 control and prevention from a One Health perspective.

Serological assays are commonly used to identify past infections and evaluate vaccine efficiency. Several serologic methods are used to determine their efficiency for detecting SARS-CoV-2 antibodies [17,18,19]. IgG-based enzyme-linked immunosorbent assays (ELISA) were found to be the most accurate for COVID-19 antibody detection at least 3 weeks after symptom onset [20]. IgG and other antibodies can still be detected several months after the initial infection due to their residual nature in the body. ELISAs can detect these antibodies for retrospective studies [21]. It should be noted that ELISA tests are not well-suited for immediate diagnoses or measuring antibodies during the acute phase of COVID-19 infections, as tests have shown to have variability [22] or low sensitivities [23]. In 2023, a seroprevalence study was conducted at the Montpellier Zoo and Sigean African Reserve in southern France. Out of 205 serum samples collected between 2021 and 2022 from 44 different species, eight were initially screened seropositive for SARS-CoV-2 by an indirect ELISA and seven were further confirmed positive by surrogate virus neutralization test (sVNT) for S protein antibodies, including the first instances of SARS-CoV-2 exposure in two species, the springbok (*Antidorcas marsupialis*) and the vicuna (*Lama vicugna vicugna*) [24]. Therefore, seroprevalence studies have the advantage in wildlife when obtaining fecal or nasal samples routinely is not possible [25,26,27,28]. Such assays could be utilized for monitoring vaccination responses by measuring virus-specific antibodies. The vaccine used by both zoological facilities in the present study was produced by Zoetis (Parsippany, New Jersey, United States), which was one of the few animal vaccines released during the peak of the pandemic in 2021 [29,30,31]. The Zoetis vaccine consists of a recombinant trimeric S protein derived from the Wuhan-Hu-1 strain [32]. Later in 2022, this vaccine was shown to be effective against the Delta variant B.1.617.2 in domestic cats [33]. In goats, it produced a substantial antibody immune response with titers peaking at approximately 42 days before tapering until day 180 [32]. However, there is a lack of published research regarding the efficiency of this vaccine in species housed in zoological institutions, as well as its efficacy against newer COVID-19 variants of concern.

This retrospective study aims to address this gap by analyzing SARS-CoV-2 seroprevalence and vaccine-induced immune responses in different captive species from Fort Wayne Zoo (Indiana) and Brookfield Zoo (Illinois) in the US.

## 2. Materials and Methods

### 2.1. Serum Samples

Two sets of serum samples from a variety of animal species housed at two zoological institutions (114 samples in Fort Wayne Zoo and 122 samples in Brookfield Zoo) were used. These serum samples were previously collected by each zoo and stored at −20 °C or −80 °C. Samples were opportunistically collected during the clinical management of the animals.

**Figure 1:**
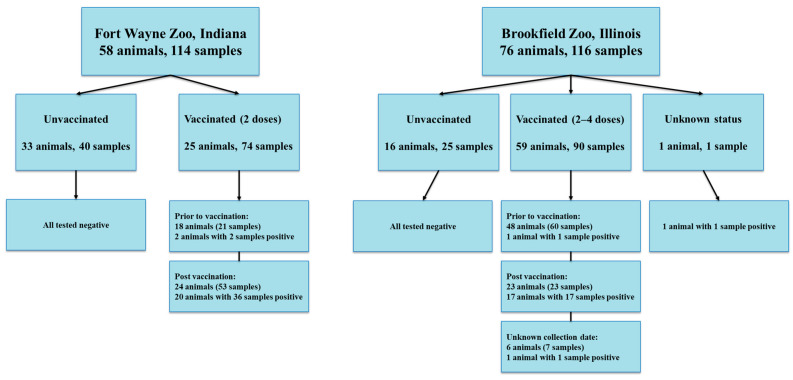
Sample information collected at Fort Wayne Zoo and Brookfield Zoo.

For Fort Wayne Zoo, 114 samples were collected from 33 unvaccinated animals (40 samples) and 25 vaccinated animals (21 samples from pre-vaccination of 18 animals and 53 post-vaccination of 24 animals) between 20 May 2020 and 14 February 2024 (Figure 1).

Out of 122 serum samples at Brookfield Zoo, six samples including one sample with unknown information for animal identification, vaccination status, and sample collection date information, one sample from an unvaccinated animal with unknown collection date, and four samples with unknown immunization status were excluded for further analysis, and the remaining 116 samples were collected from 59 vaccinated animals (60 samples prior to vaccination, 23 samples at post vaccination, 7 samples with unknown collection dates), 16 unvaccinated animals (25 samples), and 1 animal with unknown immunization status between 1 January 2015 and 9 April 2023 (Figure 1).

### 2.2. Vaccination

In this study, one of the main aims was to measure seroconversion and the duration of seropositivity in vaccinated animals of two zoological institutions. At two zoos, selected animals received a recombinant S protein vaccine regimen manufactured by Zoetis. The regimen consisted of an initial vaccine injection followed by a booster injection approximately 3 weeks later through an intramuscular route. Immunization with this S subunit vaccine would induce production of antibodies against the S protein only. A positive SARS-CoV-2 sVNT result for S antibodies in immunized animals could be due to an immune response induced by this Zoetis S protein subunit vaccine or a previous natural SARS-CoV-2 infection. Therefore, the SARS-CoV-2 nucleocapsid (N) ELISA (N-ELISA) test was utilized to further confirm whether positive animals had previous exposure, since vaccinated animals would not produce antibodies against the N protein. Serological results of all 230 samples in this study were based on the results of sVNT and N-ELISA. The minimum duration of the immune response was measured by the number of months between the last vaccine and the last positive sample.

The animals at the Fort Wayne Zoo received two doses of vaccine between early December 2021 and early January 2022, except for North American river otter-1, which received the vaccines in the fall of 2022 (Table 1).

Animals at Brookfield Zoo received the vaccine regimen in a similar timeframe (Table 2 and Table A3). A binturong and a fishing cat received a third vaccine one month after the booster (Table 2 and Table A3). A sloth bear, 2 Angolan colobus, an Amur leopard, 3 African painted dogs, an Amur tiger, 2 African lions, 3 reindeer, 2 binturongs, 2 snow leopards, a brown bear, 2 polar bears, 1 fishing cat, and a North American river otter were given a third booster at various time points. The aforementioned Amur tiger, African lions, and 1 snow leopard received a fourth vaccine in 2024 (Table A3).

A complete list of unvaccinated species from both Fort Wayne Zoo and Brookfield Zoo, and additional vaccination status for Brookfield Zoo animals, is listed in the Appendix A (Table A1, Table A3 and Table A4).

### 2.3. SARS-CoV-2 sVNT Test Kit

The SARS-CoV-2 sVNT kit (Genscript, Piscataway, NJ, USA) uses ELISA methodology to detect the presence of neutralizing antibodies against the SARS-CoV-2 S protein in serum and is a species-independent kit for testing samples of different animal species [34]. The assay was performed according to the manufacturer’s protocol. Briefly, all samples, and the negative and positive controls were diluted at a 1:10 ratio with the Sample Dilution Buffer and shaken at 750 rpm for 30 s. Horseradish peroxide-conjugated RBD (HRP-RBD) was added to each sample and control at a 1:1 ratio. After a brief vortexing, the sample with the HRP-RBD mixture was incubated for 30 min at 37 °C and 100 µL was then added to the ELISA plate wells. The plate was incubated at 37 °C for 15 min and washed 4 times with 260 µL of 1× Wash Solution. Then, 100 µL of 3,3′,5,5′-tetramethylbenzidine (TMB) solution was added to each well and incubated in the dark at room temperature for 18 min, followed by the addition of 50 µL of Stop Solution and reading the optical density (OD) value at 450 nm on the Epoch ELISA reader (BioTek, Winooski, VT, USA). The inhibition rate was calculated with the following formula:

$$\left(1-\left(\frac{Sample\;OD}{Negative\;control\;OD}\right)\right)\times 100$$

The % inhibition cutoff for positive was at or above 30% and results below 30% were considered negative.

### 2.4. SARS-CoV-2 N ELISA

The ID Screen^®^ SARS-CoV-2 Double Antigen Multi-species ELISA (ID.Vet, Grabels, France) detects the presence of antibodies against the N protein of SARS-CoV-2, which is also a species-independent kit for testing samples of different animal species [34]. The N-ELISA assay was performed according to the manufacturer’s protocol. To start, 25 µL of Dilution Buffer 13 was added to each well on the plate, followed by 25 µL of sample, positive control, or negative control. It was incubated at 37 °C for 45 min. The plate was washed 5 times with 300 µL of 1:20 diluted Wash Concentrate 20×. One hundred microliter of Conjugate 1× was added to each well, and the plate was incubated at room temperature for 30 min. The plate was washed 5 times again using the same protocol as the first wash, and 100 µL of Substrate Solution was added to each well. The plate was incubated at room temperature in the dark for 20 min, followed by adding 100 µL of Stop Solution to each well. The plate was read at 450 nm on an Epoch ELISA reader (Biotech, Winooski, VT, USA). The S/P percentage (S/P%) was calculated with the following formula:

$$\left(\frac{Sample\;OD-Negative\;control\;OD}{Positive\;control\;OD-Negative\;control\;OD}\right)\times 100$$

A sample with an S/P% at or greater than 60% was positive, between 50% and 60% was considered suspect, and any result at or below 50% was considered negative.

### 2.5. Cells and Pseudotyped Lentivirus

The following reagent was obtained through BEI Resources, NIAID, NIH: Human Embryonic Kidney Cells (HEK-293T) Expressing Human Angiotensin-Converting Enzyme 2, HEK-293T-hACE2 Cell Line, NR-52511. This cell line was cultured in Dulbecco’s Modified Eagle’s Medium (DMEM) with L-glutamine (Corning, Corning, NY, USA, MT10013CM) supplemented with 10% fetal calf serum (FCS) and 1X Pen/Strep (Cytiva Hyclone, Marlborough, MA, USA, SV30010). A HEK-293T cell line was procured from ATCC (CRL-3216) and cultured in DMEM with L-glutamine (Corning, MT10013CM) supplemented with 10% FCS and 1× Pen/Strep (Cytiva Hyclone, SV30010). Both cell lines were negative for *Mycoplasma* spp. through periodic detection.

The following reagent was obtained through BEI Resources, NIAID, NIH: SARS-Related Coronavirus 2, Wuhan-Hu-1 Spike D614G-Pseudotyped Lentiviral Kit, NR-53817. This kit was used to produce pseudotyped lentiviral particles that express the WT S gene. It contains 5 plasmids: Lentiviral backbone with Luc2 and ZsGreen genes (NR-52516), Helper plasmid for Gag-Pol (NR-52517), Tat1b (NR-52518), Rev1b (NR-52519), and a plasmid encoding codon-optimized SARS-CoV-2 S gene from Wuhan-Hu-1 (WH1) that contains a 21-amino-acid deletion at the end of the cytoplasmic tail and the D614G mutation (NR-53765). For generating viral particles expressing the Omicron LP 8.1 spike, the codon-optimized S gene in the pcDNA3.1 plasmid, a gift from Dr. Shan-lu Liu’s lab, was used with the lentiviral kit, substituting the WH1 S plasmid.

### 2.6. Neutralization Assay with Spike-Pseudotyped (S-Pseudotyped) Lentiviral Particles

Pseudotyped lentiviral particles were generated following the protocol previously described [35]. A total of 4 µg plasmid mix (2000 ng backbone, 440 ng gag-pol, 440 ng Tat1b, 440 ng Rev1b, 680 ng Spike) was transfected into the HEK-293T cells in a 100 mm dish using 12 µL TransIT-X2 (MirusBio, Madison, WI, USA, MIR6000). The transfected cells were ZsGreen imaged to check transfection efficiency. The cell culture supernatants were harvested at 48 and 72 h post-transfection. Both viral stocks were combined, aliquoted, and stored in −80 °C freezer. Viral titration was carried out in HEK-293T-hACE cells [35].

The neutralization assay was performed following the same protocol as in [35]. Briefly, 4 × 10^4^ of HEK-293T-ACE2 cells in 50 µL media were plated in a 96-well plate 12 h before the infection. In a separate 96-well preparation plate, sera samples were serially diluted in a 3-fold dilution (1:20, 1:60, 1:180, 1:540, 1:1620, 1:4860), and the total volume after dilutions was 60 µL in each well. The pseudovirus stock was first diluted with DMEM in a 1:20 ratio, and then 60 µL was added to each serum sample well. A no-serum control was prepared by adding 60 µL DMEM to the diluted virus. The virus/serum mixture was then incubated at 37 °C for 1 h. Following the removal of the culture media from the cell plate, 100 µL of the virus/serum mixture from each well of the preparation plate was added to the respective wells of the cell plate. The cells incubated with the virus only (no-serum control) or DMEM alone serve as positive and negative controls, respectively. After 48 h of incubation, the cells were measured for luciferase activity using the ONE-Glo reagent (Promega, Madison, WI, USA, E6120). Briefly, 50 µL of media was removed from all wells, leaving around 50 µL in each well. 50 µL One-Glo reagent was then added to each well and mixed gently. After incubation at room temperature for 3 min, 80 µL of cell lysates were transferred to a Corning 96-well white plate (Fisher, Pittsburgh, PA, USA, 07200722). Cytation 5 (BioTek) was used for measuring the luciferase activity following the manufacturer’s instructions. The data were analyzed with a non-linear regression fit to estimate the EC50 values after normalization to the virus-alone group (100% infectivity) with a cell-alone control (0% infectivity) using Prism 10 (GraphPad, MA, USA).

## 3. Results

### 3.1. sVNT and N-ELISA Results of Samples at Fort Wayne Zoo

**Figure 2:**
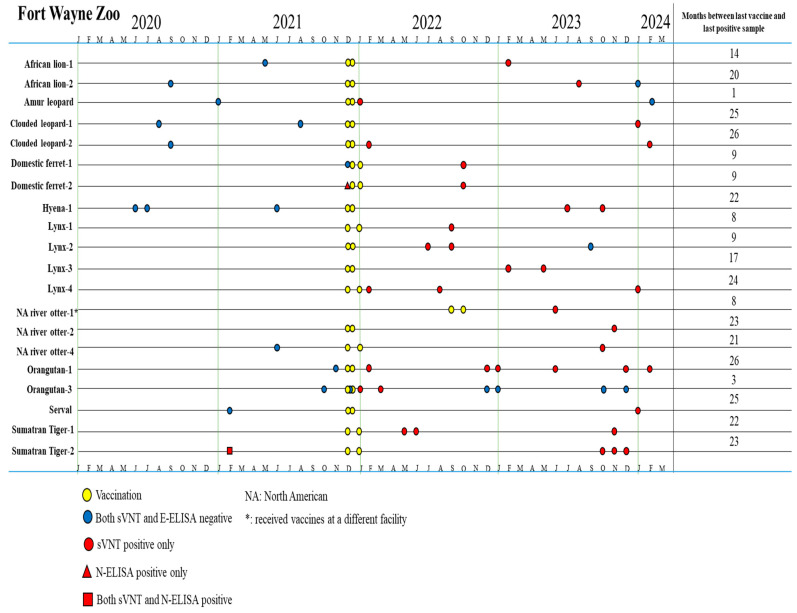
Timeline for vaccination and serum sample collection in animals that produced an immune response tested by sVNT and N-ELISA at Fort Wayne Zoo.

**Figure 3:**
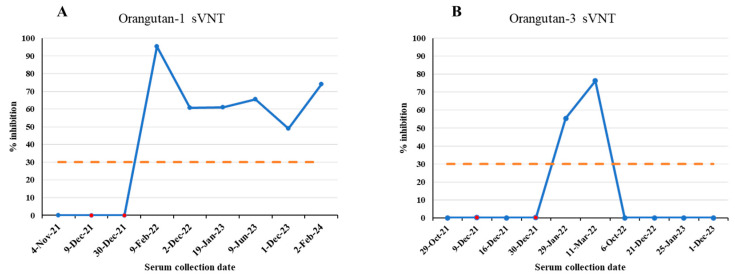
SARS-CoV-2 sVNT % inhibition results of two orangutans at Fort Wayne Zoo, Indiana. Blue dots indicate serum collection dates. Red dots indicate vaccination dates. (**A**) Orangutan-1 results. (**B**) Orangutan-3 results. Orange dash line is the 30% cutoff.

Among 114 samples of 58 animals, 36 samples collected post vaccination from 20 vaccinated animals tested positive for antibodies against the S protein of SARS-CoV-2 by the sVNT test (Table 1). Our data showed that 20 of 24 (83.3%) vaccinated animals showed seroconversion after SARS-CoV-2 immunization (Table 1). Anti-S antibodies were detected in immunized tigers (14–20 months), Amur leopard (1 month), clouded leopards (25–26 months), domestic ferrets (9 months), hyena (22 months), lynxes (8–24 months), otters (8–23 months), orangutans (3–26 months), serval (25 months), tigers (22–23 months) (Figure 2). Four vaccinated animals without seroconversion are hyena #2 and #3 and orangutan #2 and #4. The orangutans had varied immune responses to the vaccine. Orangutans #2 and #4 did not develop antibodies at any point after vaccination. Orangutan #1 developed antibodies and continued to test positive by sVNT for over 2 years. However, serological immune responses in orangutan #3 were detected for only 3 months after vaccination (Figure 2 and Figure 3). This highlights the importance of being mindful of individual serologic responses; not every animal will react the same way to a vaccine or infection.

By contrast, among 21 samples collected prior to vaccination from 18 vaccinated animals, one sample from domestic ferret #2 tested positive by N-ELISA and one sample from Sumatran tiger #2 was positive by both sVNT and N-ELISA (Table 1). The 40 samples of 33 unvaccinated animals (12 samples of 9 unvaccinated birds (ostrich, white stork, marabou stork, peahen, crane, black swan, chicken, peacock, and peahun), 16 samples of 14 unvaccinated primates (4 De Brazza’s monkeys, 4 swamp monkeys, 2 Javan gibbon, 1 capuchin, 2 colobus monkeys and 1 ring-tailed lemur), and 12 samples of 10 Carnivora animals (1 red panda, 1 bat-eared fox, 2 dingoes, 4 banded mongooses, 2 California sea lions)) were all negative by both sVNT and N-ELISA (Table A1). These data indicated that different zoo animal species had a low rate of SARS-CoV-2 previous exposure (2/51 animals, 3.9%).

Before vaccination, Sumatran tiger #2 had one positive sample by both sVNT and N-ELISA, suggesting that it had SARS-CoV-2 infection before vaccination. Concordantly, this tiger was recorded with SARS-CoV-2 infection that was also seen in Sumatran tiger #1 in early 2021. Using a real-time RT-qPCR test for fecal samples previously described [36], viral shedding occurred for 9 days in Tiger #1 and for 28 days in Tiger #2. The full data set for this infection can be found in the Appendix A (Table A2). One serum sample collected prior to vaccination for domestic ferret #2 tested positive for antibodies against the N protein, suggestive of a previous infection with SARS-CoV-2.

**Table 1:** sVNT and N-ELISA results of 74 serum samples of 25 vaccinated animals at Fort Wayne Zoo.

Species	1st Dose	2nd Dose	Date of Samples	Positive sVNT/Total Sample	Positive N-ELISA
African Lion-1	12-8-21	12-29-21	5-19-21, 2-8-23 ^S^	1/2	0
African Lion-2	12-8-21	12-29-21	9-3-20, 8-10-23 ^S^, 1-31-24	1/3	0
Amur leopard	12-8-21	12-29-21	1-24-21, 1-11-22 ^S^, 2-8-24	1/3	0
Clouded leopard-1	12-9-21	12-29-21	8-26-20, 8-11-21, 1-9-24 ^S^	1/3	0
Clouded leopard-2	12-9-21	12-30-21	9-23-20, 2-16-22 ^S^, 2-14-24 ^S^	2/3	0
Domestic ferret-1	12-14-21	1-4-22	12-10-21, 10-20-22 ^S^	1/2	0
Domestic ferret-2	12-14-21	1-4-22	12-10-21 ^N^, 10-20-22 ^S^	1/2	1
Hyena-1	12-7-21	12-28-21	6-30-20, 7-13-20, 6-8-21, 7-27-23 ^S^, 10-19-23 ^S^	2/5	0
Hyena-2	12-7-21	12-28-21	5-21-20, 8-2-23	0/2	0
Hyena-3	12-7-21	12-28-21	5-20-20, 7-5-23	0/2	0
Lynx-1	12-14-21	1-4-22	9-12-22 ^S^	1/1	0
Lynx-2	12-8-21	12-30-21	7-31-22 ^S^, 9-28-22 ^S^, 9-1-23	2/3	0
Lynx-3	12-9-21	12-29-21	2-9-23 ^S^, 5-18-23 ^S^	2/2	0
Lynx-4	12-14-21	1-5-22	2-1-22 ^S^, 8-4-22 ^S^, 1-10-24 ^S^	3/3	0
NA river otter-1 &	9-14-22	10-12-22	6-7-23 ^S^	1/1	0
NA river otter-2	12-9-21	12-30-21	11-27-23 ^S^	1/1	0
NA river otter-3	12-7-21	12-28-21	8-4-21	0/1	0
NA river otter-4	12-14-21	1-4-22	6-11-21, 10-3-23 ^S^	1/2	0
Orangutan-1	12-9-21	12-30-21	11-4-21, 2-9-22 ^S^, 12-2-22 ^S^, 1-19-23 ^S^, 6-9-23 ^S^, 12-1-23 ^S^, 2-2-24 ^S^	6/7	0
Orangutan-2	12-9-21	12-30-21	10-22-21, 6-15-22, 9-30-22, 2-21-23, 6-16-23, 12-7-23	0/6	0
Orangutan-3	12-9-21	12-30-21	10-29-21, 12-16-21, 1-29-22 ^S^, 3-11-22 ^S^, 12-21-22, 1-25-23, 10-6-23, 12-1-23	2/8	0
Orangutan-4	12-9-21	12-30-21	11-26-21, 10-26-22, 2-1-23	0/3	0
Serval	12-7-21	12-28-21	2-23-21, 1-8-24 ^S^	1/2	0
Sumatran Tiger-1	12-14-21	1-4-22	5-12-22 ^S^, 6-2-22 ^S^, 11-1-23 ^S^	3/3	0
Sumatran Tiger-2	12-14-21	1-4-22	2-25-21 ^S,N,P^, 10-16-23 ^S^, 11-1-23 ^S^, 12-19-23 ^S^	4/4	1

NA river otter: North American river otter; ^S^: samples tested positive for S-protein antibodies by sVNT; ^N^: samples tested positive for N-protein antibodies by N-ELISA; &, received vaccines at another facility; ^P^: real-time RT-qPCR tested positive for SARS-CoV-2.

### 3.2. sVNT and N-ELISA Results of Samples at Brookfield Zoo

Out of 116 serum samples collected from 76 animals, 90 samples were from 59 vaccinated animals, with 60 samples (30 January 2015 to 28 June 2021) collected prior to vaccination from 48 animals, 23 post-vaccination samples from 23 animals, and 7 samples with unknown collection dates from 6 animals. Among 59 vaccinated animals, 23 animals each had one sample collected post vaccination (14 animals also had one sample collected before vaccination and 3 animals with one sample with unknown collection date, and 6 animals only had one post vaccination sample), and remaining 36 vaccinated animals only had a total of 50 samples with 46 samples collected before vaccination (30 January 2015 to 28 June 2021) and 4 samples with unknown collection dates.

**Figure 4:**
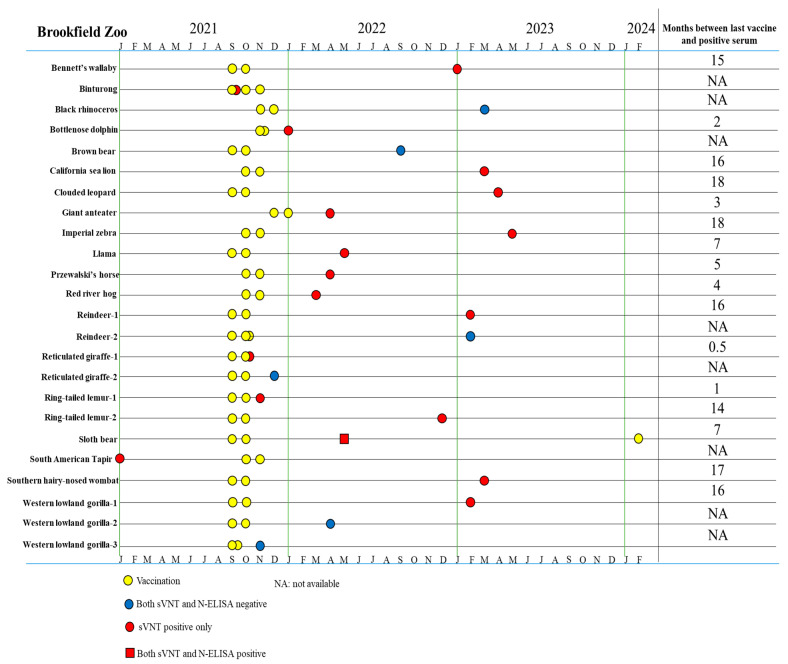
Timeline for vaccination and serum sample collection in animals that produced a positive/negative serologic result of 23 vaccinated animals with post-vaccination serum samples and one sVNT positive serum sample from prior immunization at Brookfield Zoo.

A total of 20 animals tested positive by sVNT, with one positive sample from each animal (Table 2, Figure 4). Seventeen of 23 (73.9%) vaccinated animals with samples collected post vaccination tested positive by sVNT. Vaccine-induced anti-S antibodies were detected in immunized Bennetts’ wallaby (15 months), bottlenose dolphin (2 months), California sea lion (16 months), clouded leopard (18 months), giant anteater (3 months), imperial zebra (18 months), llama (7 months), Przewalski’s horse (5 months), red river hog (4 months), reindeer (16 months), reticulated giraffe (0.5 month), ring-tailed lemurs (1–14 months), sloth bear (7 months), southern hairy-nosed wombat (17 months), and western lowland gorilla (16 months) (Figure 4). Notably, six animals were vaccinated but did not produce an immune response: two western lowlands gorillas, one reindeer, one black rhinoceros, one brown bear, and one reticulated giraffe (Table 2, Figure 4).

**Table 2:** sVNT and N-ELISA results of 23 serum samples collected at post-vaccination dates, 5 samples with unknown collection dates, and 15 samples collected from prior vaccination of 26 animals at Brookfield Zoo.

Species	1st Dose	2nd Dose	3rd Dose	Date of Sample Collection	Positive sVNT/Total Sample	Positive N-ELISA
Bennett’s Wallaby	9-16-21	10-7-21	-	4-6-17, 1-17-23 ^S^	1/2	0
Binturong ^P^	9-8-21	10-29-21	11-29-21	9-30-21 ^S^	1/1	0
Bottlenose dolphin	11-12-21	11-30-21	-	1-12-22 ^S^, NA	1/2	0
California sea lion	10-5-21	7-Nov-21	-	3-20-23 ^S^	1/1	0
Clouded leopard	9-7-21	12-Oct-21	-	7-19-19, 4-6-23 ^S^	1/2	0
Giant anteater	12-21-21	10-Jan-22	-	1-10-17, 4-9-22 ^S^	1/2	0
Imperial zebra	10-24-21	21-Nov-21	-	6-5-15, 5-3-23 ^S^	1/2	0
Llama	9-14-21	9-Oct-21	-	4-18-18, 5-27-22 ^S^	1/2	0
North American river otter	NA	NA	NA	NA ^S^	1/1	0
Przewalski’s horse	10-17-21	11-21-21	-	6-23-16, 4-8-22 ^S^	1/2	0
Red river hog	10-19-21	11-5-21	-	3-24-22 ^S^, NA	1/2	0
Reindeer-1	9-14-21	10-17-21	-	2-13-23 ^S^, NA	1/2	0
Reticulated giraffe-1	9-14-21	10-7-21	-	3-23-16, 10-29-21 ^S^	1/2	0
Ring-tailed lemur-1	9-14-21	10-8-21	-	12-28-16,11-16-21 ^S^	1/2	0
Ring-tailed lemur-2	9-15-21	10-11-21	-	10-1-14, 12-13-22 ^S^	1/2	0
Sloth bear	9-9-21	10-10-21	2-21-24	4-3-19, 5-18-22 ^S,N^	1/2	1
South American tapir	10-23-21	11-21-21	-	1-20-21 ^S^	1/1	0
Southern hairy-nosed wombat	10-28-21	11-16-21	-	3-1-23 ^S^	1/1	0
Western grey kangaroo	10-25-21	11-16-21	-	NA ^S^	1/1	0
Western lowland gorilla-1	9-9-21	10-5-21	-	3-12-19, 2-1-23 ^S^	1/2	0
Black rhinoceros	11-17-21	12-5-21	-	3-9-23	0/1	0
Brown bear	9-13-21	10-16-21	-	5-13-19, 9-13-22	0/2	0
Reindeer-2	9-20-21	10-12-21	10-17-21	2-13-23	0/1	0
Reticulated giraffe-2	9-17-21	10-7-21	-	2-4-16, 12-21-21	0/2	0
Western lowland gorilla-2	9-9-21	10-2-21	-	1-29-19, 4-15-22	0/2	0
Western lowland gorilla-3	9-8-21	9-30-21	-	11-15-21	0/1	0

-: did not received vaccine; NA: not available; ^S^: samples tested positive for S-protein antibodies by sVNT; ^N^: samples tested positive for N-protein antibodies by N-ELISA; ^P^: real-time RT-qPCR tested on feces positive for SARS-CoV-2 on 9-28-21 [36].

Among 60 samples collected prior to vaccination of 48 vaccinated animals, only one from a South American tapir tested positive by the sVNT assay (Table 2 and Table A3, Figure 4), indicating that this tapir had previous exposure to SARS-CoV-2. The remaining two animals that had samples tested positive by sVNT were a river otter (unknown immunization status) and a vaccinated kangaroo (unknown sample collection date). The 25 samples of 16 different animals were all negative by sVNT (Table A4). The same set of 116 samples was tested further by N-ELISA and only one 1 sample collected post vaccination from a sloth bear was positive which was also positive by sVNT, suggestive of a previous infection by SARS-CoV-2.

### 3.3. Neutralization Assay with S-Pseudotyped Lentiviral Particles

**Figure 5:**
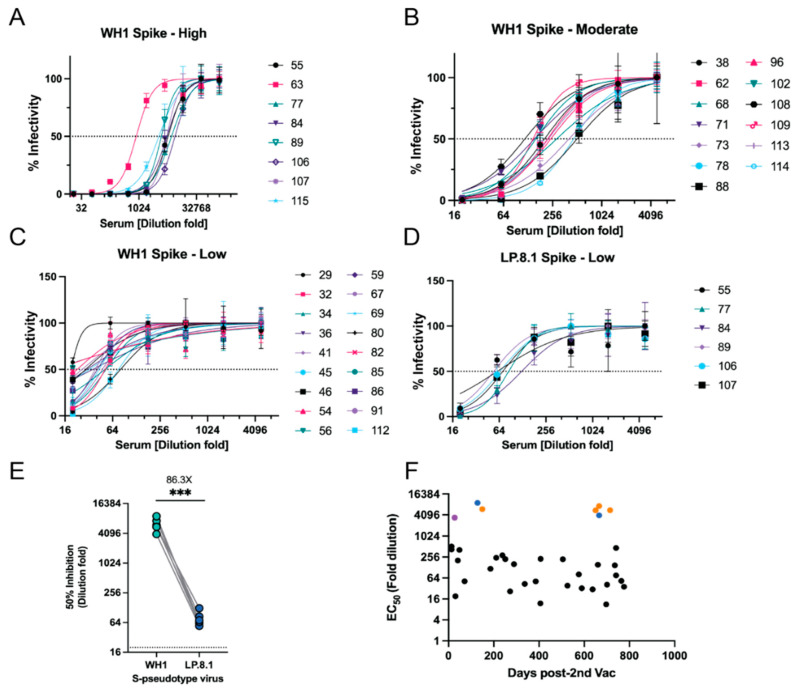
Neutralization assay with S-pseudotyped lentiviral particles. (**A**–**C**) Serum samples with high, moderate, and low neutralizing titers against the WH1-S pseudotype virus, respectively. (**D**) Serum samples with high, moderate, and low neutralizing titer against LP.8.1-S pseudotype virus. (**E**) Neutralizing activity comparison of sera against two pseudotype viruses. Student t test; ***, *p* < 0.001. (**F**) Neutralizing titers (EC50 values) with respect to the time (Days post-2nd vaccination) of serum samples collected. Orange and blue dots: Sumatran tiger #1 and #2, respectively; purple dot: Lynx.

To quantitatively evaluate protective antibody levels in the serum samples, we performed viral entry assays with S-pseudotyped viral particles to measure neutralizing antibody titers. This assay served as a powerful tool to evaluate the vaccine efficiency against SARS-CoV-2 infection during the COVID-19 pandemic [37,38,39]. To this end, we generated two S-pseudotyped lentiviruses: one displays the S protein of the Wuhan-1 (WH1) strain, and the other one presents the S protein of a contemporary Omicron LP.8.1 strain. Among the 42 samples tested from animals at Fort Wayne Zoo, 36 samples showed neutralization activity against the WH1-S pseudovirus with various titers. Two methods, sVNT vs. S-pseudotyped viral neutralization assay, have a similar positive detection rate (Chi-square test, *p* = 0.3539). Of the 42 samples from animals at Fort Wayne Zoo, eight samples showed high neutralizing titers with estimated EC_50_ values ranging from 874 to 7343 (dilution-fold) (Figure 5A and Table A5). Thirteen and 18 samples had moderate and low neutralizing titers with EC_50_ values of 117–464 and 11–81 (dilution-fold), respectively (Figure 5B,C). We then selected 17 samples from the high (n = 8) and moderate (n = 9) groups to test against the LP.8.1-S pseudovirus. Among the 17 samples, 6 from the high group showed relatively low neutralizing activity with estimated EC_50_ values ranging from 55–124 dilution-fold (Figure 5D). By comparing their EC50s against the WH1-S pseudovirus, there was an average of 86.3-fold reduction (53.83–118.8 of 95% CI, *p* = 0.0003) in neutralizing activity (Figure 5E). The rest of the selected samples showed no neutralizing activity (Table A5). This result suggests that the WH1-S-based vaccine may not provide protective immunity against newer Omicron variants, which have been reported in human serological studies [40,41,42]. The detailed results of this assay can be found in Appendix A Table A5. We also analyzed the neutralizing titers with respect to the time when the serum samples were collected after the second dose of vaccination. We found that the majority of the samples had a relatively low neutralizing titer (<256 dilution-fold), even within six months post-vaccination (Figure 5F). Interestingly, samples from Sumatran tigers (orange and blue dots) and Canada Lynx (purple) exhibited high neutralizing titers (Figure 5F). Of note, one Sumatran tiger (orange dots) was sampled multiple times after 2nd vaccination and had high neutralizing titers even after two years of vaccination. This was not expected as serological studies in vaccinated humans reported that vaccine-induced antibodies typically significantly decreased within 6–8 months [43]. Given that tigers can be naturally infected by SARS-CoV-2 and the S protein vaccine cannot prevent mucosal infection, we reason that the high titer after two years of vaccination may be due to an immunization boost from later natural infections.

## 4. Discussion

Given the ability of SARS-CoV-2 to infect multiple animal species and its potential for zoonotic transmission, it is crucial to track its spread in settings where humans and animals are in frequent close contact. Retrospective studies like the current study are valuable for analyzing outbreaks, identifying new susceptible hosts or reservoirs, and assessing serologic responses to vaccination. Serological screening is a widely utilized method for detecting prior SARS-CoV-2 infections and evaluating vaccine-induced immunity in large-scale seroprevalence studies.

Throughout 2020, studies predicted that various mammalian species might be susceptible to SARS-CoV-2 [44,45,46,47]. One study compared 25 key residues in human ACE2 (hACE2) to those in 410 other species that could potentially serve as reservoir hosts [44]. Based on this study, animals in the Primates order such as Sumatran orangutans and western lowland gorillas were predicted to have very high susceptibility due to their ACE2 protein matching all 25 key residues. Interestingly, despite being predicted to have low susceptibility [44], a few species such as the white rhinoceros [48] and two new species from the present study, South American tapir and sloth bear, were positive for SARS-CoV-2. This highlights that SARS-CoV-2 adaptation to new hosts could be ascribed to other non-ACE2 factors including individual immune responses and viral entry through receptors other than ACE2.

This current study marks the first documented case of SARS-CoV-2 exposure in a sloth bear, making it the second species within the Ursidae family to contract the virus. In 2024, an Asian black bear (*Ursus thibetanus*) tested positive for the Omicron variant BF.7.15 during an outbreak at a zoological institute [49]. The serum sample of the sloth bear tested positive for both S-protein and N-protein antibodies following vaccination, indicating a wild-type infection (Figure 4). To date, SARS-CoV-2 has not been detected in other Ursidae family members, such as grizzly, brown, or American black bears [28,50,51]. One study using the online SWISS-MODEL platform predicted reduced susceptibility in polar bears due to a deletion in ACE2 residues 28–36 [52]. This region includes amino acids crucial for RBS-ACE2 binding, and its absence may hinder viral entry, though this hypothesis has not yet been tested in vivo. The detection of SARS-CoV-2 in a sloth bear underscores the importance of routine serum collection in zoological institutions. As long as the virus continues to circulate among animals, the emergence of new host species remains a concern, especially in environments housing diverse species in close proximity.

The South American tapir was determined to have a wild-type SARS-CoV-2 infection, as evidenced by the presence of S-protein antibodies before vaccination (Figure 4). Aforementioned susceptibility simulations predicted members of the *Tapiridae* family have low susceptibility to SARS-CoV-2 due to low ACE2 homology with hACE2 [44]. During the COVID-19 pandemic, a study reported the detection of a bovine-like coronavirus in two Indonesian tapirs with enteric illness [53]. Phylogenetic analysis revealed the betacoronavirus infecting the tapirs had multiple changes to the S1 and S2 proteins, indicating that the virus can adapt to new hosts through mutations, similar to that of SARS-CoV-2. As with other animals such as dogs, cats, and cattle, tapirs might be susceptible to both SARS-CoV-2 and other coronaviruses. However, as with bears, there is limited research exploring the relationship between SARS-CoV-2 and species within the *Tapiridae* family.

Domestic ferret-2 from Fort Wayne Zoo was positive for N-protein antibodies and negative for S-protein antibodies prior to vaccination, suggesting that the ferret had antibodies from a prior infection or was experiencing asymptomatic infection at the time of serum collection, as the ferret was noted to be in good health. Wild-type SARS-CoV-2 infections in companion ferrets have been documented in the US and Slovenia [2,54]. Experimental studies have also shown that ferrets can transmit the virus to other ferrets through direct contact and airborne routes [55]. Interestingly, a previously mentioned susceptibility study noted ferrets may have medium susceptibility due to a reduced number of key ACE2 residues necessary for RBD binding [47].

Both Sumatran tigers from Fort Wayne Zoo tested positive in feces by real-time RT-PCR for COVID-19 on 2 February 2021. Viral shedding of tiger-1 occurred for 9 days, and viral shedding of tiger-2 occurred for 28 days (Table A2). This correlates with the S-protein and N-protein positive sample from Sumatran tiger-2 on 25 February 2021 (94.59% inhibition, 66.14% S/P, respectively). The Sumatran tigers in Fort Wayne Zoo had a long immune response to the vaccine, lasting over 22 months. Longer duration of serologic responses in tigers may be a result of both the initial infection and follow-up immunization, in support of a previous finding in humans that infection-acquired immunity boosted with vaccination produced longer immune responses than vaccination only [56].

As COVID-19 has continued to spread, several variants of concern have emerged [57]. Because of this, it may be necessary to continue providing susceptible species with routine booster shots. Select animals at the Brookfield Zoo were given a third and fourth vaccine, while animals at Fort Wayne Zoo only received the initial 2-dose regimen due to restriction of vaccination availability. The Felid Taxon Advisory Group recommends giving boosters every 6–12 months after the initial two-dose vaccination in nondomestic felines [58]. This is especially necessary due to past evidence of reverse zoonosis occurring between humans and both domestic and nondomestic felines [8,11,12]. With both parties receiving routine vaccinations, it lowers the chance of a feline host becoming a reservoir for new SARS-CoV-2 variants and in turn reinfecting humans. Expanding seroprevalence studies can address this concern for new reservoir hosts outside of known families, such as mink and white-tailed deer [59,60]. Mink hosts were seen during the first year of the pandemic in Denmark [61]. A mutation of nucleotide C25936T was found in all mink farms within Denmark and subsequently in the COVID-19 genome of afflicted mink farm workers. This mutation did not derive from humans, as it was not present in the human population prior to 10 June 2020. However, in the weeks following the mink-to-human transmission, mutations were found in 40% of human SARS-CoV-2 cases in the population near the farms. A combination of zoonosis and reverse zoonosis was responsible for several mutations in minks and humans throughout Denmark and the Netherlands during this time [16,61].

This study has several limitations. First, it is a retrospective study, and the sample collection lacks continuity as serum samples were opportunistically collected. Collection of blood samples requires anesthesia for many species, limiting the ability to conduct broad surveys in a more controlled manner. Thus, serum samples were not routinely collected after vaccination. At the Brookfield Zoo, for instance, only one post-vaccination sample was collected, limiting the analysis of the antibody responses. Second, the overall sample sizes, both in total number of animals and by species, were small. Future serosurveillance studies should implement routine serum collection to improve vaccination distribution strategies and monitor the risk of COVID-19 outbreaks more effectively.

SARS-CoV-2 continues to impact humans and animals five years after its emergence in humans. This retrospective serosurveillance study discovered wild-type SARS-CoV-2 exposure in sloth bears and South American tapirs for the first time and reinforced the trend of tiger susceptibility. Regular vaccinations can produce a substantial immune response in several species, and by continuing this practice in zoological institutions, they could help decrease the emergence of new variants. Continued serosurveillance within zoological institutions, domestic companion animals, and wildlife will aid in the analysis of continued SARS-CoV-2 transmission between animals and humans. This will help to minimize the development of new strains or mutations and virus transmission through zoonosis and reverse zoonosis.

## Data Availability

All data related to the current study is provided in the manuscript.

## Appendix A

**Table A1:** 40 negative serum samples of 33 unvaccinated animals tested by sVNT and N-ELISA at Fort Wayne Zoo.

Order	Family	Species	Number of Negative Sera/Animal Number	Date of Sample Collection
Anseriformes	*Anatidae*	Black swan	1/1	6-14-22
Aves	*Ciconiidae*	White stork	1/1	7-19-20
Aves	*Gruidae*	Wattled crane	1/1	12-2-21
Aves	*Phasianidae*	Domestic chicken	1/1	12-21-21
Aves	*Phasianidae*	Indian peafowl	3/3	12-3-22, 6-24-23, 12-21-23
Aves	*Struthionidae*	Ostrich	4/1	7-31-20, 4-5-21, 3-9-22, 8-21-24
Carnivora	*Ailuridae*	Red panda	1/1	5-24-21
Carnivora	*Canidae*	Bat-eared fox	1/1	1-20-22
Carnivora	*Canidae*	Dingo	4/2	2-18-21, 7-18-23, 10-26-23, 12-27-23
Carnivora	*Herpestidae*	Banded mongoose	4/4	2-22-21, 9-9-22, 9-9-22, 6-15-23
Carnivora	*Otariidae*	California sea lion	2/2	12-4-22, 12-15-23
Ciconiiformes	*Ciconiidae*	Marabou stork	1/1	7-1-21
Primates	*Cebidae*	Capuchin monkey	1/1	7-2-20
Primates	*Cercopithecidae*	Colobus monkey	2/2	6-11-20, 8-29-23
Primates	*Cercopithecidae*	De Brazza’s monkey	4/4	1-27-21, 7-19-21, 8-9-21, 12-5-23
Primates	*Cercopithecidae*	Swamp monkey	5/4	10-6-20, 1-14-21, 5-27-21, 6-15-21, 9-14-21
Primates	*Hylobatidae*	Javan gibbon	3/2	9-22-20, 3-15-21, 9-25-23
Primates	*Lemuridae*	Ring-tailed lemur	1/1	10-3-20

**Table A2:** SARS-CoV-2 real-time RT-PCR results of fecal samples for both Sumatran tiger 1 and 2 at Fort Wayne Zoo. Real-time RT-PCR used as previously described [36].

Month	Date	N1 Ct		N2 Ct	
		Tiger-1	Tiger-2	Tiger-1	Tiger-2
February 2021	2	&	&	30.97	22.77
	3	26.36	25.57	26.15	24.12
	4	29.95	27.47	28.73	27.04
	5	30.43	25.04	29.61	25.54
	6	32.05	30.51	30.55	29.92
	7	38.45	33.74	36.84	32.33
	8	34.31	27.09	32.85	26.43
	9	36.00	27.30	37.78	26.17
	10	40.00	31.61	38.30	30.99
	11	40.00	20.00	40.00	18.64
	12	40.00	-	40.00	-
	13	40.00	31.00	40.00	29.13
	14	40.00	33.81	40.00	32.40
	15	39.87	26.54	40.00	25.28
	16	40.00	-	40.00	-
	17	40.00	30.68	40.00	29.32
	18	40.00	31.45	40.00	31.00
	19	40.00	24.41	40.00	24.19
	20	40.00	33.68	40.00	32.44
	21	39.18	33.34	40.00	32.43
	22	40.00	-	40.00	-
	23	40.00	31.78	40.00	30.47
	24	40.00	31.73	36.00	30.39
	25 *	40.00	32.57 *	40.00	31.12 *
	26	40.00	28.09	40.00	26.88
	27	40.00	26.44	40.00	25.96
	28	40.00	25.36	40.00	24.56
March 2021	1	40.00	26.05	40.00	25.24
	2	40.00	40.00	40.00	40.00
	3	40.00	40.00	40.00	40.00
	4	40.00	40.00	40.00	40.00
	5	40.00	40.00	40.00	40.00
	6	40.00	40.00	40.00	40.00
	7	40.00	40.00	40.00	40.00
	8	40.00	40.00	40.00	40.00
	9	40.00	40.00	40.00	40.00

Symbols: &, N1 was not tested; -, serum was not collected; *, date of positive ELISA sample. Notes: Ct values of N1 and N2 real-time RT-PCR are provided. Ct value of RT-qPCR below 40 was positive. Ct values of the samples with negative results were set to 40.00.

**Table A3:** A total of 48 samples from 34 vaccinated animals tested negative by both sVNT and N-ELISA at Brookfield Zoo. Among these 48 samples, 45 samples were collected prior to vaccination and 3 samples had unknown collection dates.

Order	Family	Species	Sample Number/ Animal Number	Date of Vaccination	Date of Sample Collection
Artiodactyla	*Bovidae*	Red-flanked duiker	1/1	9-19-21, 10-10-21	10-5-20
Artiodactyla	*Cervidae*	Reindeer	3/2	9-17-21, 10-17-21, 2-13-24	8-15-18, 8-15-18, 5-16-20
Artiodactyla	*Giraffidae*	Okapi	2/1	10-1-21, 10-22-21	11-13-18, 1-13-20
Carnivora	*Canidae*	African painted dog	5/5	9-27-21, 10-19-21, 2-18-24 10-2-21, 10-23-21 10-2-21, 10-23-21, 2-18-24 9-16-21, 10-23-21, 2-18-24 10-2-21, 10-23-21	4-30-20 3-11-21 3-11-21 3-11-21 3-11-21
Carnivora	*Mustelidae*	North American river otter	1/1	9-15-21, 10-09-21, 3-06-24	3-15-19
Carnivora	*Otariidae*	California sea lion	4/2	10-16-21, 11-7-21 9-27-21, 11-7-21	9-24-19, 9-18-20 3-6-18, 9-18-20
Carnivora	*Phocidae*	Grey seal	1/1	10-5-21, 11-7-21	5-5-21
Carnivora	*Ursidae*	Brown bear	1/1	9-13-21, 10-16-21, 3-04-24	5-2-19
Carnivora	*Ursidae*	Polar bear	3/2	9-13-21, 10-4-21, 2-8-21 9-13-21,10-4-21, 2-22-24	2-12-21 NA, NA
Carnivora	*Viverridae*	Binturong	3/2	9-8-21, 11-6-21, 11-23-21 9-7-21, 9-27-21, 9-16-24	12-10-20, NA 7-1-19
Carnivora	*Felidae*	African lion	2/2	9-8-21, 11-7-21, 11-29-21 9-8-21, 11-30-21, 12-21-21	3-17-20 3-17-20
Carnivora	*Felidae*	Amur leopard	3/2	9-8-21, 10-28-21, 11-20-21 9-8-21, 11-11-21, 11-29-21	6-29-17, 3-3-21 1-8-20
Carnivora	*Felidae*	Amur tiger	1/1	9-16-21, 10-26-21, 11-23-21, 2-21-24	6-18-20
Carnivora	*Felidae*	Fishing cat	3/2	9-9-21, 11-15-21, 12-5-21 9-7-21, 10-16-21	10-22-17, 12-2-20 5-13-21
Carnivora	*Felidae*	Snow leopard	3/2	9-8-21, 10-28-21, 11-20-21, 2-21-24 9-8-21, 11-11-21, 11-29-21	6-29-17, 3-3-21 1-8-20
Perissodactyla	*Equidae*	Przewalski’s horse	2/1	10-24-21, 11-11-21	6-20-19, 6-28-21
Perissodactyla	*Rhinocerotidae*	Black rhinoceros	2/1	10-26-21, 11-30-21	1-5-17, 5-27-21
Perissodactyla	*Tapiridae*	South American tapir	1/1	10-23-21, 11-12-21	10-6-20
Primates	*Atelidae*	Black-handed spider monkey	1/1	9-20-21, 10-11-21	5-21-19
Primates	*Cercopithecidae*	Allen’s swamp monkey	2/1	9-10-21, 10-2-21	5-11-15, 8-27-20
Primates	*Cercopithecidae*	Angolan colobus	2/1	9-10-21, 10-28-21, 2-9-24	10-6-16, 2-10-21
Primates	*Cercopithecidae*	Red-tailed guenon	2/1	9-21-21, 10-18-21	1-30-15, 1-13-20

NA: Not available.

**Table A4:** A total of 25 samples from 16 unvaccinated animals tested negative by sVNT and N-ELISA at Brookfield Zoo.

Order	Family	Species	Sample Number/Animal Number	Date of Sample Collection
Artiodactyla	*Bovidae*	Nigerian dwarf goat	2/1	2-10-16, 9-1-21
Artiodactyla	*Delphinidae*	Bottlenose dolphin	2/1	1-28-19, 12-29-21
Carnivora	*Otariidae*	California sea lion	2/2	10-11-16, 11-4-20
Carnivora	*Phocidae*	Grey seal	4/2	9-7-17, 11-18-20, 2-15-19, 11-18-20
Carnivora	*Felidae*	African lion	2/1	10-28-16, 1-2-20
Carnivora	*Felidae*	Clouded leopard	1/1	3-8-20
Carnivora	*Felidae*	Fishing cat	1/1	10-13-20
Carnivora	*Viverridae*	Binturong	1/1	2-22-17
Diprotodontia	*Macropodidae*	Bennett’s wallaby	1/1	3-23-23
Diprotodontia	*Vombatidae*	Southern hairy-nosed wombat	1/1	10-19-20
Pilosa	*Myrmecophagidae*	Giant anteater	2/1	7-7-20, 8-1-17
Primates	*Cercopithecidae*	Red-tailed guenon	4/2	6-19-19, 10-1-20 3-30-19, 11-13-20
Primates	*Hylobatidae*	White-cheeked gibbon	2/1	9-30-15, 9-24-20

**Table A5:** Neutralization assay results with Spike-pseudotyped lentiviruses.

Species (Animal #)	Sample ID.	Sample Collection Days Post-2nd-Vac	EC50 (WH1-Spike)	EC50 (LP.8.1-Spike)	Fold Reduction
Sumatran tiger (#1)	106	128	9048 (H)	65.73	137.7
	89	666	3965 (H)	54.6	72.6
Sumatran tiger (#2)	63	−313	873.7 (H)	ND	NA
	55	149	5985 (H)	60.68	98.6
	107	650	5540 (H)	71.41	77.6
	77	666	7343 (H)	84.76	86.6
	84	714	5561 (H)	124.5	44.7
Lynx (#1)	62	251	222.9 (M)	ND	NA
Lynx (#2)	108	185	117.2 (M)	NT	NA
	67	272	26.35 (L)	NT	NA
Lynx (#3)	113	407	226.4 (M)	ND	NA
	78	505	221.1 (M)	ND	NA
Lynx (#4)	115	27	3423 (H)	ND	NA
	96	211	242.9 (M)	NT	NA
	109	735	147.5 (M)	NT	NA
Hyena (#1)	80	576	80.85 (L)	NT	NA
	71	660	153.6 (M)	ND	NA
Hyena (#2)	70	−586	ND	NT	NA
Orangutan (#1)	38	41	204 (M)	NT	NA
	45	337	43.21 (L)	NT	NA
	34	385	51.31 (L)	NT	NA
	41	526	38.6 (L)	NT	NA
	36	701	41.16 (L)	NT	NA
	85	764	52.93 (L)	NT	NA
Orangutan (#2)	48	274	ND	NT	NA
Orangutan (#3)	29	30	19.05 (L)	NT	NA
	32	71	51.59 (L)	NT	NA
	46	391	29.21 (L)	NT	NA
Orangutan (#4)	47	398	ND	NT	NA
Clouded leopard (#1)	60	−140	ND	NT	NA
	112	741	76.01(L)	NT	NA
Clouded leopard (#2)	73	48	407.4 (M)	ND	NA
	86	776	35.92 (L)	NT	NA
Amur leopard	88	13	511.1 (M)	ND	NA
African lion (#1)	54	406	11.96 (L)	NT	NA
African lion (#2)	69	589	32.5 (L)	NT	NA
River otter (#1)	68	238	287.2 (M)	ND	NA
River otter (#2)	56	697	11.21 (L)	NT	NA
River otter (#3)	82	−146	22.83 (L)	NT	NA
River otter (#4)	59	637	30.31 (L)	NT	NA
Domestic Ferret (#1)	102	289	159.7 (M)	NT	NA
Serval	114	741	464.3 (M)	ND	NA

Note: ND, not detectable; NT, not tested; NA, not applicable; H: High, M: medium, L: Low inhibition.
